# Detection of Novel QTLs Regulating Grain Size in Extra-Large Grain Rice (*Oryza sativa* L.) Lines

**DOI:** 10.1186/s12284-016-0109-2

**Published:** 2016-07-25

**Authors:** Shuhei Segami, Tatsuya Yamamoto, Katsuyuki Oki, Tomonori Noda, Hiroyuki Kanamori, Harumi Sasaki, Satomi Mori, Motoyuki Ashikari, Hidemi Kitano, Yuichi Katayose, Yukimoto Iwasaki, Kotaro Miura

**Affiliations:** 1 Fukui Prefectural University, Faculty of Biotechnology, 4-1-1 Kenjojima, Matsuoka, Eiheiji-cho, Yoshida-gun, Fukui 910-1195 Japan; 2 Japan Society for the Promotion of Science, Chiyoda-ku, Tokyo, 102-8472 Japan; 3 Bioscience and Biotechnology Center, Nagoya University, Furo-cho, Chikusa, Nagoya, Aichi 464-8601 Japan; 4 Agrogenomics Research Center, National Institute of Agrobiological Sciences, 2-1-2 Kannondai, Tsukuba, Ibaraki 305-8602 Japan

**Keywords:** QTL, Grain size, Rice (*Oryza sativa* L.)

## Abstract

**Background:**

Grain size is an important trait that affects rice yield. Although many genes that contribute to grain size have been cloned from mutants or by quantitative trait locus (QTL) analysis based on bi-parental mapping, the molecular mechanisms underlying grain-size determination remain poorly understood. In this study, we identified the lines with the largest grain size and detected novel QTLs affecting the grain size.

**Results:**

We screened the National Institute for Agrobiological Sciences Genebank database and identified two rice lines, BG23 with the widest grain and LG10 with the longest grain. Using these two lines, we performed QTL analysis for grain size. Eight QTLs were detected during the QTL analyses using F_2_ populations derived from crosses between the large-grain lines BG23 or LG10 and the middle-size grain cultivars Nipponbare and Kasalath. Both BG23 and LG10 possessed large-grain alleles of four major QTLs: *GW2*, *GS3*, *qSW5/GW5*, and *GW8*. Other three minor QTLs were derived from BG23. However, these QTLs did not explain the differences in grain size between these two lines. Additionally, four QTLs for grain length or width were detected in an F_2_ population derived from a cross between BG23 and LG10; this population lacked the strong effects of the four major QTLs shared by both parent plants. Of these newly detected QTLs, the effects of two QTLs, *GL3b* and *GL6*, were confirmed by progeny testing. Comparison of the length of inner epidermal cells in plants homozygous for BG23 and LG10 alleles indicated that *GL3b* and *GL6* genes regulate cell elongation and cell division, respectively.

**Conclusions:**

In this study, we detected 12 loci including 14 QTLs regulating grain size from two lines with largest grains available in Japanese stock. Of these loci, we confirmed the effect of two gene loci and mapped their candidate region. Identification of novel genes regulating grain size will contribute to our understanding of the molecular mechanisms controlling grain size.

**Electronic supplementary material:**

The online version of this article (doi:10.1186/s12284-016-0109-2) contains supplementary material, which is available to authorized users.

## Background

Grain size is an important trait in breeding, serving as a factor determining rice yield. Generally, genes regulating grain size are classified into two groups, those controlling cell elongation and those controlling cell division. A short-grain phenotype due to shortened cell length in the loss-of-function mutants of brassinosteroid (BR)-related gene *D61* implies that these genes control cell elongation (Segami et al. 2012). Furthermore, *SRS3* and *SRS5* are also involved in cell elongation. *SRS3* and *SRS5* encode a kinesin-13 protein, and an α-tubulin, respectively (Kitagawa et al. 2010, Segami et al. 2012). These *srs* mutants commonly show short-grain phenotypes due to shortened cell lengths, similar to the BR-related mutants. However, the *Srs5* mutant is seemingly not involved in BR signal transduction. *D1* and *TUD1* are involved in cell division (Izawa et al. 2010; Hu et al. 2013). The mutants *d1* and *tud1* commonly show a short-grain and dwarf phenotype owing to reduced cell numbers. *D1* and *TUD1* encode the α-subunit of a heterotrimeric G-protein and U-box ubiquitin ligase, respectively, and *d1* is epistatic to the *tud1* mutation (Ashikari et al. 1999; Fujisawa et al. 1999; Hu et al. 2013). Of these mutants, we succeeded in classifying *srs3, Srs5, d1,* and *d61* into cell elongation- or division-type mutants by comparing the cell length and cell number of the inner-epidermal cells of lemma (Izawa et al. 2010; Kitagawa et al. 2010; Segami et al. 2012). Quantitative trait locus (QTL) analysis also revealed other genes involved in grain size regulation. *GS3* controls grain length and weight (Fan et al. 2006; Mao et al. 2010; Takano-Kai et al. 2009); *GW2* and *qSW5/GW5* control grain width and weight (Shomura et al. 2008; Song et al. 2007; Weng et al. 2008); *GS5* controls grain width, filling, and weight (Li et al. 2011); *GW8* controls grain width (Wang et al. 2012); *GL3.1* (also named *OsPPKL1*) controls grain length (Qi et al. 2012; Zhang et al. 2012); *TGW6* and *GW6a* control grain weight (Ishimaru et al. 2013; Song et al. 2015); and *GL7/GW7* controls grain length and width (Wang et al. 2015a, b). Interestingly, among these genes cloned by QTL analysis, only *GL7/GW7* was involved in cell elongation, whereas the others were involved in cell division. It is noteworthy that these genes encode a variety of different proteins involved in various signal transduction pathways. *GS3* encodes the γ-subunit 3 of a heterotrimeric G-protein (Li et al. 2012); *GW2* encodes a RING-type domain with E3 ubiquitin ligase activity (Song et al. 2007); *qSW5/GW5* encodes a novel nucleoprotein that interacts with polyubiquitin in a yeast two-hybrid experiment (Weng et al. 2008); *GS5* encodes a putative serine carboxypeptidase and acts as a positive regulatory factor in the cell cycle (Li et al. 2011); *GW8* encodes the transcription factor Squamosa promoter-binding protein-like 16 containing the *miR156*-targeted site (Wang et al. 2012); *GL3.1* encodes a Ser/Thr phosphatase of the protein phosphatase of the kelch-like family (Qi et al. 2012; Zhang et al. 2012); *TGW6* encodes a protein with indole-3-acetic acid-glucose hydrolase activity (Ishimaru et al. 2013); *GW6a* encodes a functional GCN5-related *N*-acetyl-transferase-like protein that harbors intrinsic histone acetyl-transferase activity (Song et al. 2015); and *GL7/GW7* encodes a homolog of the *Arabidopsis thaliana* TONNEAU1 recruiting motif (TRM) protein (Wang et al. 2015a, b). Although many genes were cloned and identified, the molecular mechanisms underlying grain size determination are not well understood, because most of these genes are not involved in the same signal transduction pathways. To clarify these mechanisms, it is necessary to identify and classify additional genes that contribute to grain size, whether through cell elongation or cell division, by unified methodology. Therefore, the aim of this study was to identify, from the National Institute for Agrobiological Sciences (NIAS) Genebank database (http://www.gene.affrc.go.jp), the rice lines with the largest grain, and using these lines, detect novel QTLs and elucidate how these QTLs control grain size.

## Results

### BG23 and LG10 Display the Extra-Large Grain Phenotype

**Fig. 1:**
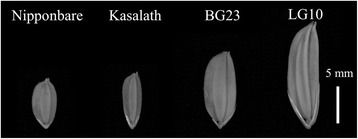
Grain size and morphology of middle-size grain cultivars Nipponbare and Kasalath and extra-large grain lines BG23 and LG10. Bar = 5 mm

The parental lines used in QTL analysis for grain size were selected by screening for the largest-grain rice lines in the public database NIAS Genebank. The grain width and length of screened lines ranged from 1.7 to 4.6 mm and 3.4 to 14.3 mm, respectively (Additional file 1: Figure S1A and B). Of these screened lines in NIAS Genebank, BG23 (grain width 4.6 mm) and LG10 (grain length 14.3 mm), which produced seeds with the largest width and length, respectively, were selected as parental lines for QTL analysis. Because the grain length of Nipponbare and Kasalath was 6.97 ± 0.28 mm and 7.99 ± 0.33 mm, respectively, and near to the median value of the NIAS Genebank database, we used these lines as middle-size lines for grain length and width comparison (Fig. 1, Table 1). The grain length of BG23 and LG10 was 11.24 ± 0.42 mm and 14.25 ± 0.46 mm, respectively (Fig. 1, Table 1). The grain width of BG23 and LG10 was 4.43 ± 0.22 mm and 4.06 ± 0.36 mm, respectively, whereas that of Nipponbare and Kasalath was 3.27 ± 0.27 mm and 2.55 ± 0.21 mm, respectively (Fig. 1, Table 1). Because the grains of BG23 and LG10 were significantly larger compared to those of the middle-size grain cultivars, these lines were expected to possess different composition of genes regulating grain size. Clarifying the compositions and effects of each gene will provide information about epistasis and effect of interactions of these genes. Therefore, this analysis was expected to provide novel and useful genes with the potential to increase grain yield by controlling grain size.

**Table 1:** Comparisons of grain length and width in rice lines Nipponbare, Kasalath, BG23, and LG10

Traits	Nipponbare	Kasalath	BG23	LG10
Grain length (mm)	6.97 ± 0.28	7.99 ± 0.33	11.24 ± 0.42	14.25 ± 0.46
Grain width (mm)	3.27 ± 0.27	2.55 ± 0.21	4.43 ± 0.22	4.06 ± 0.36

Student’s t-test was used to generate the *P* values. An asterisk indicates a statistically significant difference from the neighboring value (*P* < 0.01). Significant difference of grain length was detected between Nipponbare and BG23, Kasalath and BG23, Nipponbare and LG10, and BG23 and LG10 (*P* < 0.01). Significant difference of grain width was detected between Nipponbare and BG23, Kasalath and BG23, and Nipponbare and LG10 (*P* < 0.01). The difference of grain width detected between BG23 and LG10 was not significant (*P* > 0.05)

### BG23 and LG10 Shared Four Major QTLs for Grain Size

**Fig. 2:**
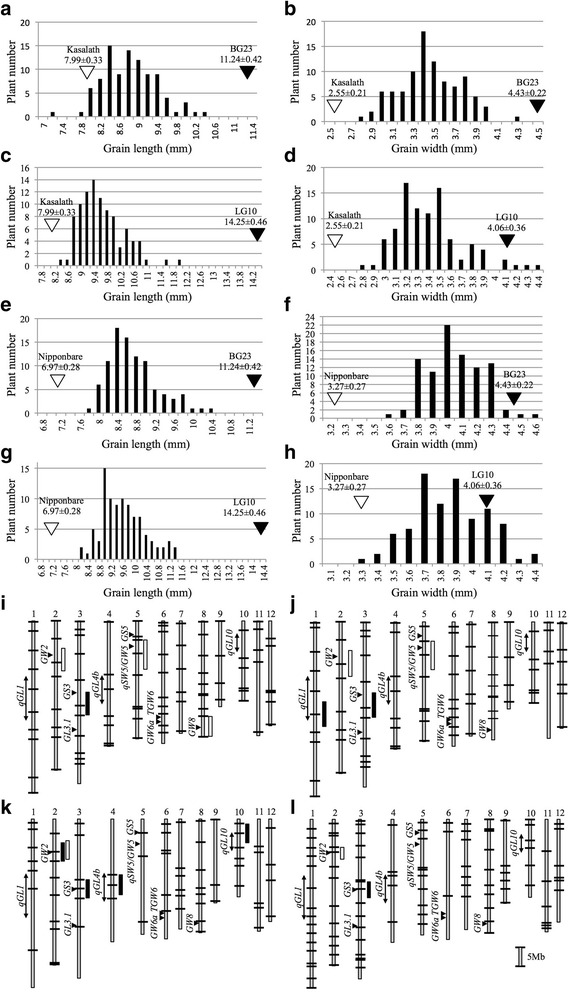
Frequency distribution of grain length and width and quantitative trait loci (QTLs) detected in F_2_ populations. Frequency distribution of grain length is shown in **a**, **c**, **e**, and **g**. Frequency distribution of grain width is shown in **b**, **d**, **f**, and **h**. F_2_ populations derived from Kasalath × BG23 (**a** and **b**), Kasalath × LG10 (**c** and **d**), Nipponbare × BG23 (**e** and **f**), and Nipponbare × LG10 (**g** and **h**) crosses. Vertical white and black arrowheads indicate the means of the middle-size grain cultivars and extra-large grain lines, respectively. Genetic linkage maps of F_2_ populations derived from Kasalath × BG23 (**i**), Kasalath × LG10 (**j**), Nipponbare × BG23 (**k**), and Nipponbare × LG10 (**l**). Boxes indicate positions of regions harboring QTLs with the logarithm of the odds > 3.0 for grain length (black) and width (white). Horizontal black arrowheads indicate the position of previously cloned QTLs for grain size. Double-headed arrows indicate the position of previously detected QTLs for grain size. Bars indicate marker positions

To identify the genes regulating grain length and width in BG23 and LG10, four combinations of F_2_ populations were derived from the following crosses: Kasalath × BG23, Kasalath × LG10, Nipponbare × BG23, and Nipponbare × LG10. A plot of grain length and width of the F_2_ individuals in each population showed a continuous distribution (Fig. 2a–h), indicating that the differences in grain traits between the extra-large grain lines and middle-size grain lines were regulated by QTLs. QTL analysis for grain length and width detected two loci on Chr2 and Chr3 that were shared by all four F_2_ populations (Fig. 2i–l, Table 2, Additional file 2: Figure S2A–H) and one locus on Chr5 shared by two F_2_ populations derived from Kasalath crosses (Fig. 2i and j, Table 2). Unshared QTLs were detected on Chr1, Chr4, Chr10, and Chr8 (Fig. 2i–k, Table 2). In total, we detected eight QTLs including seven loci in this analysis (Fig. 2i–k, Table 2).

**Table 2:** Quantitative trait loci for grain length and width detected in F_2_ populations derived from the crosses Kasalath × BG23, Kasalath × LG10, Nipponbare × BG23, and Nipponbare × LG10

Populations	Traits	Chr^a^	Source	Marker interval	LOD^b^	Additive	Dominance	PVE^c^
Kasalath × BG23 F_2_	Grain length	3	BG23	RM5551 -RM5488	5.22	0.44	−0.46	20.28
	Grain width	2	BG23	RM7581 -RM6639	7.43	0.25	0.00	28.27
		5	BG23	TG93 -d1-8	4.02	0.18	−0.06	17.88
		8	BG23	RM3452 -RM3155	5.2	0.15	−0.16	18.54
Kasalath × LG10 F_2_	Grain length	1	LG10	B1075D06 -RM3143	3.93	0.44	0.03	17.41
		3	LG10	RM5551 -RM5488	6.39	0.60	−0.59	27.36
	Grain width	2	LG10	RM7581 -RM6639	11.12	0.30	−0.10	41.15
		5	LG10	TG93 -d1-8	6.47	0.23	−0.06	27.16
Nipponbare × BG23 F_2_	Grain length	2	BG23	RM5654 -RM1313	5.71	0.39	0.06	24.38
		3	BG23	RM3033 -GS3-PstI	16.82	0.58	−0.44	60.39
		4	BG23	RM2565 -RM5714	3.55	0.33	0.07	15.87
		10	BG23	RM5271 -RM5147	3.34	0.18	0.36	15.07
	Grain width	2	BG23	RM5654 -GW2-HinfI	14.49	0.20	−0.04	46.39
Nipponbare × LG10 F_2_	Grain length	3	LG10	RM3033 -RM2346	14.99	0.71	−0.42	51.99
	Grain width	2	LG10	RM5654 -RM1313	25.59	0.27	−0.04	71.48

^a^Chromosome number. ^b^log10 of the odds ratio. ^c^Phenotypic variance explained

As several QTLs were detected around previously cloned gene loci, we performed marker analysis for the genes *GW2, GS3, qSW5/GW5*, and *GW8*. Both BG23 and LG10 possessed all of the alleles responsible for large grain size in all four genes (Additional file 3: Figure S3A–H). The QTLs for grain width and length, detected on Chr2 and Chr3, respectively, in all tested populations correspond to *GW2* and *GS3*, whereas the QTLs for grain width detected on Chr5 and Chr8 correspond to *qSW5/GW5* and *GW8*.

The QTLs for grain length on Chr4 and Chr10 detected from the Kasalath × LG10 cross and Chr1 detected from the Nipponbare × BG23 cross include *qGL1, qGL4b,* and *qGL10*; loci have been previously reported (Kato et al. 2011; Wang et al. 2012; Yan et al. 2014) (Fig. 2j and k). In total, we detected seven loci including four cloned and three non-cloned QTLs for grain size. However, as *GW8* was detected only in the F_2_ population derived from the Kasalath × BG23 cross, these minor QTLs appeared to be masked by the effects of the major QTLs. Here, we examined whether the QTL analysis for grain length using F_2_ population derived from BG23 and LG10 cross is possible to detect the same loci on Chr1, Chr4, and Chr10 detected in other combinations of F_2_ populations and whether these loci can fully explain the differences between BG23 and LG10. Hence, the QTLs for grain length and width were investigated using the F_2_ population derived from the BG23 × LG10 cross, thus avoiding the strong effects of the four QTLs shared by the parents.

### Novel QTLs Detected from the BG23 × LG10 Cross

Screening for simple sequence repeat (SSR) markers for QTL analysis that could indicate the differences in genotypes between BG23 and LG10 was unsuccessful, because these two parental lines exhibited a highly similar banding pattern on the gel. To construct the linkage map, we performed re-sequencing of the parents and designed BeadArray markers using detected single nucleotide polymorphisms (SNPs).

**Fig. 3:**
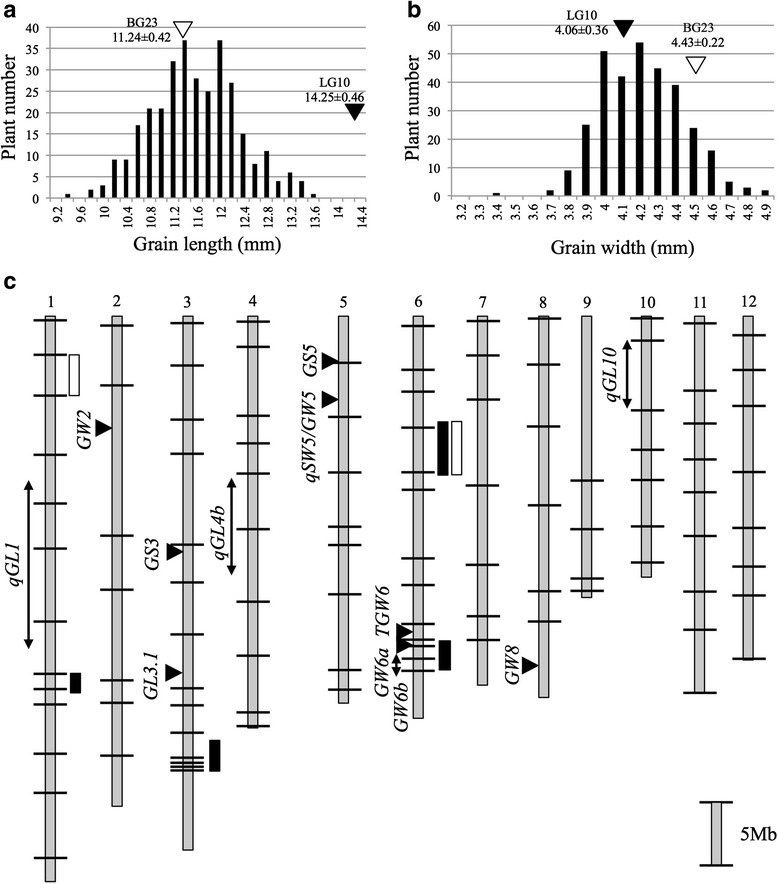
Frequency distributions of grain length and width and quantitative trait loci (QTLs) detected in F_2_ populations derived from the BG23 × LG10 cross. Frequency distributions of grain length (**a**) and width (**b**). Vertical white and black arrowheads indicate the means for BG23 and LG10, respectively. (**c**) Boxes indicate the positions of regions harboring QTLs with the logarithm of the odds > 3.0 for grain length (black) and width (white). Horizontal black arrowheads indicate the positions of previously cloned QTLs for grain size. Double-headed arrows indicate the positions of previously detected QTLs for grain size. Bars indicate marker position

The histograms of grain length and width in F_2_ individuals showed a continuous distribution, indicating that the differences between BG23 and LG10 with respect to these parameters were likely regulated by multiple QTLs (Fig. 3a and b). QTL analysis of the F_2_ population from the BG23 × LG10 cross revealed four QTLs for grain length on Chr1, Chr3, the short arm of Chr6 (Chr6S), and the long arm of Chr6 (Chr6L) and two QTLs for grain width on Chr1 and Chr6S (Fig. 3c, Table 3, and Additional file 3: Figure S3I and J). Additionally, the QTL for grain length on Chr6L included *GW6a* and *GW6b* (Song et al. 2015) (Fig. 3c). Overall, we detected six QTLs including five loci (Fig. 3c, Table 3). The QTL for grain width on Chr1 and that for grain length on Chr3 and Chr6S represent novel loci regulating grain size. A two-dimensional scan revealed no significant epistatic interactions among these QTLs in this population (Additional file 4: Figure S4).

**Table 3:** Quantitative trait loci for grain length and width detected in F_2_ population derived from the BG23 × LG10 cross

Populations	Traits	Chr^a^	Source	Marker interval	LOD^b^	Additive	Dominance	PVE^c^
BG23 × LG10 F_2_	Grain length	1	LG10	aa01009984 -BGLG_1-29.92_Hinf	4.16	0.26	−0.07	5.59
		3	LG10	BGLG_3-36.1_Pst -aa03002747	9.52	0.36	−0.18	12.85
		6	LG10	ac06000397 -ab06001036	5.40	0.27	−0.23	7.44
		6	LG10	bglg6_27 -BGLG_6-28.12_Hae	20.04	0.51	0.06	24.73
	Grain width	1	LG10	aa01004844 -ab01000509	5.80	0.10	−0.01	7.77
		6	LG10	ac06000397 -ab06001036	11.74	0.12	−0.10	15.13

^a^Chromosome number. ^b^log10 of the odds ratio. ^c^Phenotypic variance explained

### Phenotypic Evaluation of Novel QTLs

**Fig. 4:**
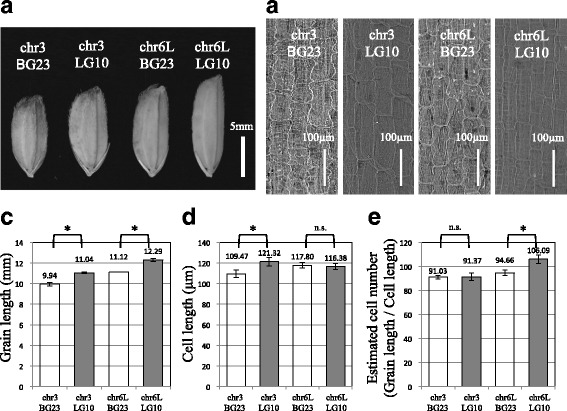
Comparison of grain length and inner-epidermal cell length in segregation lines. **a** Grain morphology of Chr3-BG23, Chr3-LG10, Chr6-BG23, and Chr6-LG10. Bar = 5 mm. **b** Inner-epidermal cells in the lemma of Chr3-BG23, Chr3-LG10, Chr6-BG23, and Chr6-LG10. Bars = 100 μm. **c** Grain length of Chr3-BG23, Chr3-LG10, Chr6-BG23, and Chr6-LG10. **d** Cell length of Chr3-BG23, Chr3-LG10, Chr6-BG23, and Chr6-LG10. **e** Estimated cell number of Chr3-BG23, Chr3-LG10, Chr6-BG23, and Chr6-LG10. Numbers in **c**–**d** indicate the averages. Student’s t-test was used to generate the *P* values. * indicates statistical significance from the neighboring value (*P* < 0.05). n.s. indicates no significance (*P* > 0.05)

To confirm the effect of detected QTLs on grain length, we screened F_3_ lines including one segregating locus and three other fixed loci. Of the four loci, we screened two segregation lines for QTLs detected on Chr3 and Chr6L (Additional file 5: Figure S5) and from these segregation lines selected plants homozygous for BG23 and LG10 alleles on Chr3 (Chr3-BG23 and Chr3-LG10) and Chr6L (Chr6L-BG23 and Chr6L-LG10). The grain length of Chr3-LG10 and Chr6L-LG10 were significantly longer compared to those of Chr3-BG23 and Chr6L-BG23, respectively (Fig. 4a, c). These results clearly indicate that these two loci include genes responsible for grain length. LG10 alleles on Chr3 and Chr6L have a positive effect on grain length. We named these two genes *GL3b* and *GL6*. To determine whether these grain-size regulating genes are involved in cell division or cell elongation, we compared the length of the inner epidermal cell in lemma by using scanning electron microscopy (SEM) (Fig. 4b). The length of the inner epidermal cells was significantly longer in Chr3-LG10 than in Chr3-BG23, whereas no significant difference was observed between Chr6L-BG23 and Chr6L-LG10 (Fig. 4d). In contrast, the estimated number of cells in Chr6L-LG10 was significantly higher than that in Chr6L-BG23 (Fig. 4e). These results indicate that *GL3b* and *GL6* affect cell elongation and cell division, respectively.

### Genetic Mapping of *GL3b* and *GL6*

**Fig. 5:**
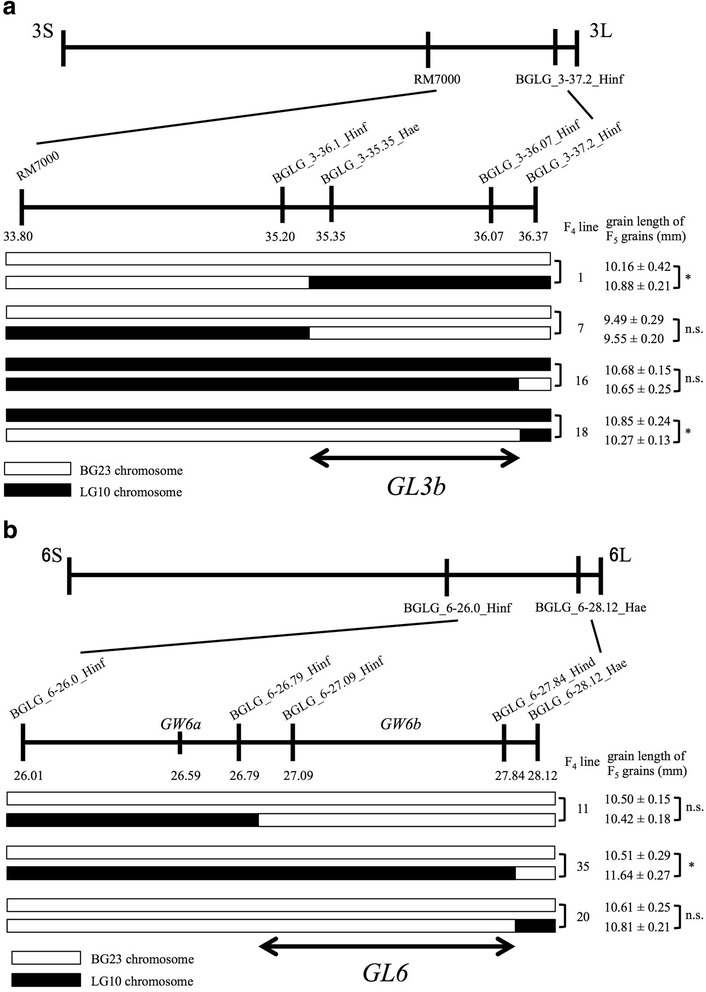
Genetic mapping of *GL3b* and *GL6*. **a** Recombinant plants possessing breakpoint at the Chr3 locus were screened from the F_3_ population of segregation lines by using PCR markers RM7000 and BGLG_3-37.2_Hinf. We found four F_3_ recombinant plants. Because the F_3_ plants were heterozygous, we selected F_4_ plants homozygous for BG23 or LG10 chromosome and recombinant chromosome to compare the phenotype for grain length. At least three homozygous plants were selected for each genotype. The grain length of these homozygous F_4_ plants was measured by using 50 F_5_ seeds. Two types of genotype selected among homozygous plants from each line are indicated to the left of the F_2_ line number. The grain length of each line is indicated to the right of the F_2_ line number. The results of the *t*-test for grain length between the plants with or without recombination are indicated to the right of the grain length of F_5_ grains. *GL3b* gene locus was mapped into a 1.2-Mb region between BGLG_3-36.1_Hinf and BGLG_3-37.2_Hinf. **b** Recombinant plants possessing breakpoint at the Chr6L locus were screened from the F_3_ population of segregation lines by using PCR markers BGLG_6-26.0_Hinf and BGLG_6-28.12_Hae. We found three F_3_ recombinant plants. The grain length of homozygous F_4_ plants was measured by using 50 F_5_ seeds. *GL6* gene locus was mapped into a 1.3-Mb region between BGLG_6-26.79_Hinf and BGLG_6-28.12_Hae. White and black boxes indicate BG23 and LG10 chromosomes, respectively. Student’s *t*-test was used to generate the *P* values. * indicates statistical significance from the neighboring value (*P* < 0.05). n.s. indicates no significance (*P* > 0.05). The numbers under the marker name indicate marker’s position (Mb)

To narrow down the candidate region of *GL3b* and *GL6*, we performed genetic mapping using recombinant lines of each locus. Recombinant screening of these two loci using F_3_ population of segregating lines resulted in four and three recombinants in *GL3b* and *GL6* regions, respectively (Fig. 5a and b). Because the F_3_ plants possess heterozygous chromosomes, we selected F_4_ plants homozygous for BG23 or LG10 and recombinant chromosome of each *GL3b* and *GL6* region to compare the phenotype of grain length. Using the data on grain length in F_5_ seeds obtained from selected F_4_ plants from each line, we performed mapping analysis for *GL3b* and *GL6* gene loci.

At the *GL3b* locus, we screened recombinant plants with a recombination breakpoint between the PCR markers RM7000 and BGLG_3-37.2_Hinf and obtained four F_3_ recombinant plants (Fig. 5a). These plants were grown to obtain F_4_ seeds, which were then sawn and the resulting plants were screened homozygous for BG23 or LG10 chromosome as well as for recombinant chromosomes and compared the grain length of the resulting F_5_ seeds. The recombinant plant selected from F_4_ line no. 1 with a recombination event between 35.20 Mb and 35.35 Mb showed a significantly longer grain compared to that of sibling plants homozygous for BG23, indicating that the causal gene of *GL3b* is located on the right from 35.20 Mb (Fig. 5a). Similarly, the F_4_ line no. 7 also indicates that *GL3b* is located to the right from 35.20 Mb because grain length of the recombinant plant is the same as that in plants homozygous for BG23. Similarly, the right border of *GL3b* was indicated at 36.37 Mb according to F_4_ lines 16 and 18 (Fig. 5a). Taken together, we concluded that *GL3b* locus is located in a region 1.2 Mb long, between 35.20 Mb and 36.37 Mb on Chromosome 3.

At the *GL6* locus, we screened three recombinant plants possessing recombination breakpoint between the PCR markers BGLG_6-26.79_Hinf and BGLG_6-28.12_Hae (Fig. 5b). Because the recombinant plant selected from the F_4_ line no. 1 with a recombination event between 26.79 Mb and 27.09 Mb shows same overlap in grain length with sibling plants homozygous for BG23, we conclude that the causal gene *GL6* locates to the right from 26.79 Mb (Fig. 5b). Similarly, the right border of *GL6* was indicated as 28.12 Mb by F_4_ lines 36 and 20 (Fig. 5b). Taken together, we concluded that *GL6* locus locates in a 1.3-Mb long region, between 26.79 Mb and 28.12 Mb on Chromosome 6.

## Discussion

In this study, we detected eight QTLs on seven loci from four populations derived from crosses between large-grain lines and middle-size grain lines. Furthermore, we newly detected six QTLs on five loci from the population derived from the cross between large grain lines. In total, we detected 12 loci including four cloned QTL genes. Both BG23 and LG10 contained four major grain-size regulating genes: *GW2, GS3, qSW5/GW5,* and *GW8* (Fig. 2i-l). The results presented herein indicate that the additive effect of *GW2* locus for grain width, and of *GS3* locus for grain length, detected from four combinations of F_2_ population were distributed from 0.2 to 0.3 and from 0.44 to 0.7, respectively (Fig. 2i-l, Table 2). These additive effects are consistent with previously reported effects of *GW2* and *GS3* (Fan et al. 2006; Song et al. 2007). The dominant *GS3* locus detected in our four populations was distributed from −0.42 to −0.59. This indicates that plants heterozygous for *GS3* locus show equal phenotype as plants homozygous for functional *GS3* gene. Thus, the *GS3* locus detected in our study behaves in a recessive manner, which is consistent with previous report (Fan et al. 2006). The locus *qSW5/GW5* was detected in the F_2_ population derived from Kasalath × BG23 and Kasalath × LG10 crosses (Fig. 2i and j, Table 2). Originally, *qSW5* was detected from descendants derived from the Kasalath × Nipponbare cross (Shomura et al. 2008), with the Nipponbare allele of *qSW5* being recessive. Our results corroborate this feature of *qSW5. GW8* was detected only in the F_2_ population derived from the cross between BG23 × Kasalath, whereas LG10 also contained large grain allele (Fig. 2i, Additional file 3: Figure S3D). The *GW8* locus detected in the BG23 × Kasalath cross showed additive effect of 0.15 and dominance effect of −0.16 (Table 2). This result indicates that the large grain allele of *GW8* locus behaves in a recessive manner, although previously *GW8* was reported as semi-dominant gene (Wang et al. 2012). Because the effects of *GW8* locus for grain size regulation are comparatively weaker compared to the effects of the other three genes and not detected in the BG23× Nipponbare cross, the effects of *GW8* seemed to be masked or distorted by other three major QTLs. Therefore, the effects of minor QTLs may not be detectable. Although it is difficult to compare the effects of QTLs detected from different populations because the genetic background affects the effect of each QTL, we assumed that there should be some alternative QTLs explain the differences between BG23 and LG10. Thus, we tried to detect QTLs for grain size between BG23 and LG10 while avoiding the four major QTLs. As we expected, we could detect additional four QTLs in the F_2_ population derived from the cross between BG23 and LG10. Of these four QTLs, two loci located on Chr3 and Chr6L and named *GL3b* and *GL6* were confirmed to be involved in cell elongation and cell division, respectively (Fig. 4a-e). Observation of the inner epidermal cells in lemma revealed that *GL3b* promotes grain length by promoting cell elongation (Fig. 4b-e). Using the same method, the genes responsible for cell elongation had been identified from a BR-related mutant (*d61*) and microtubule-related mutants (*srs3* and *Srs5*) (Kitagawa et al. 2010; Segami et al. 2012). TRM-containing protein (*GL7/GW7*) cloned in the QTL analysis also regulates cell elongation in rice (Wang et al. 2015b). Because these studies cloned only those genes involved in cell elongation, genetic diversity of the grain size in rice was considered to have developed mainly owing to the differences in the genes responsible for cell division. Therefore, clarifying the molecular functions of *GL3b* via cloning and genetic analysis of these genes will be important in elucidating the complex genetic mechanism underlying grain size regulation. *GL6* is involved in cell division (Fig. 4b-e). Since it was detected in the F_2_ population derived from the crossing of BG23 and LG10, both of which share the long-grain alleles of *GS3* and *GW8* (Fig. 3c, Additional file 3: Figure S3)*, GL6* promotes grain length in an additive manner with *GS3* and *GW8*. This indicates that *GL6* belongs to a genetically separated signal transduction pathway from those of *GS3* and *GW8*.

In contrast, the grain of BG23 is wider than the grain of LG10 (Fig. 1, Table 1). Nevertheless, LG10 provided positive additive effects for all detected QTLs for grain width in the F_2_ population derived from the crossing of BG23 and LG10. This is explained by the grain length of LG10, which is too long in the longitudinal direction to fill grain hull. Therefore, starch input is not sufficient to fill the volume of the grain hull. This starch shortage leads to unfilled and thinner grains than predicted by the genetic potential of LG10.

## Conclusion

In this study, we detected 12 loci that are involved in the regulation of grain size, using rice lines with largest grain size in Japanese stock (Figs. 2i-l, 3c). Among these loci, we identified two loci *GL3b* and *GL6* that promote grain length through increased cell length and cell division, respectively (Fig. 4b-e). Although we confirmed the effects of the two loci detected in this study, the effects of six loci remain unknown (Fig. 2i-l, and Fig. 3c). The results indicate that the molecular mechanisms underlying grain size determination are complex. To clarify the mechanisms and genetic architecture, we need to isolate the genes and test the genetic interactions of each gene in future studies.

## Methods

### Plant Materials

Two rice (*Oryza sativa* L.) cultivars, Nipponbare (*japonica*) and Kasalath (*indica*), and two extra-large grain rice lines, BG23 (*japonica*) and LG10 (*japonica*), were used in this study. BG23 (accession number: 8966) and LG10 (accession number: 84410) were obtained from NIAS Genebank after screening for lines with the widest and the longest grain, omitting the data with input errors (containing wrong trait annotations, low reliability of the reads, etc.).

For the QTL analysis, five F_2_ populations were produced from the following parental crosses: Kasalath × BG23, Kasalath × LG10, Nipponbare × BG23, Nipponbare × LG10, and BG23 × LG10. Phenotypic evaluations for QTL analysis using these F_2_ populations were performed in the 2012 growing season. Phenotypic evaluations for F_2_, F_3,_ and F_4_ populations derived from BG23 × LG10 cross were performed in the 2013, 2014, and 2015 growing seasons, respectively. Rice seeds were sown at the beginning of April, and at the beginning of May the seedlings were transplanted into a paddy field at the Research Center for Bioresources Development, Fukui Prefectural University, Fukui, Japan, and grown under natural conditions.

### Phenotypic Evaluation

Harvested rice seeds were air-dried and stored in a seed dryer for at least 2 weeks. Fully filled grains were then evaluated for grain length and width. Fifty grains were randomly selected from each plant and scanned with a scanner (GT8300, EPSON, Nagano, Japan) to obtain images that were then used to measure each size using Smart Grain software (Tanabata et al. 2012).

The inner-epidermal cells of lemmas in mature grain hull were observed by SEM (TM3030, Hitachi, Tokyo, Japan). We measured cell length of the central cell layer of the hull (*n* > 20).

### QTL Analysis

Genomic DNA was extracted from each F_2_ progeny. The number of individuals and markers used from each F_2_ population for each genotype were as follows: 94 individuals and 92 markers in Kasalath × BG23 F_2_ population, 94 individuals and 90 markers in Kasalath × LG10 F_2_ population, 94 individuals and 66 markers in Nipponbare × BG23 F_2_ population, 94 individuals and 105 markers in Nipponbare × LG10 F_2_ population, and 318 individuals and 106 markers in BG23 × LG10 F_2_ population (Additional file 6: Table S1 and Additional file 7: Table S2). PCR-based markers, including SSR markers (McCouch et al. 2002; Ware et al. 2002; http://www.gramene.org/) and cleaved amplified polymorphic sequence (CAPS) markers (Konieczny and Ausubel 1993), were used to construct linkage maps. An Illumina GoldenGate Assay with VeraCode (San Diego, CA, USA) was used according to the manufacturer’s instructions.

QTL analysis was performed using the R/qtl software (http://www.rqtl.org; Broman et al. 2003). Putative QTLs were detected by using the interval mapping function. Two QTLs of the same chromosome were confirmed by composite interval mapping function. The QTLs were assumed present when the logarithm of the odds score was 3.0 or higher. The additive and dominant effects and the phenotypic variance explained by each QTL at the maximum LOD score were estimated by using the sim.geno, makeqtl, fitqtl, and effectplot functions in the R/qtl software (Broman et al. 2003).

### Marker Analysis

*GW2*-specific CAPS markers were amplified using the primers GW2-Hinf1-F and GW2-Hinf1-R. *GS3* marker analysis was carried out according to the protocol described by Fan et al. (2009) using primers SF28-U and SF28-L. *qSW5/GW5* marker analysis was conducted according to the method presented in Weng et al. (2008) using primers Indel2-F and Indel2-R, and *GW8* marker analysis was carried out using indel markers to detect a 10-bp deletion in the promoter region (Additional file 8: Table S3).

### Resequencing Analysis of the Extra-Large Grain Lines

Construction of the shotgun library and sequencing were conducted with a Genome Analyzer-IIx (GA-IIx; Illumina, USA). Rice genomic DNA was fragmented by Covaris (Woburn, MA, USA). The size-selected DNA (average length 300 bp) was purified with an AmpureXP (Beckman Coulter, Inc., Fullerton, CA, USA) and gel extraction. The standard short-read library was built using a TruSeq DNA Sample Prep Kit v2-SetA (Illumina, USA) and TruSeq SBS v5 (Illumina, USA) according to manufacturer’s instructions for sequencing runs at 2 × 100 bp total. After sequencing, GA-IIx real time analysis 1.13.48.0 and CASAVA 1.8.2 (Illumina, USA) were utilized for base calling.

The rice genome IRGSP sequence (IRGSP Build5 Pseudomolecules of the Rice Genome, http://rgp.dna.affrc.go.jp/E/IRGSP/Build5/build5.html) was used for reference mapping. The pair-ended reads were used for reference mapping with BWA ver.0.6.1 (Li and Durbin 2010) and SAMtools ver. 0.1.18 (Li et al. 2009), converting the mapped data to a SAM file and then generating a pileup of the sequence data. SNP, deletions, and insertions were detected using CLC Genomics Workbench v6.5.1 (CLCbio, Qiagen, Aarhus, Denmark). After selecting the nucleotide variations with frequency equal to 100.00, we identified 252,343 SNPs and INDELs between BG23 and Nipponbare and 326,351 SNPs and INDELs between LG10 and Nipponbare. SNPs included in the center of single-copied 60-bp sequence were selected to design a marker by running a BLAST search of the Rice Annotation Project Database (http://rapdb.dna.affrc.go.jp/).

## Additional files

Additional file 1: Figure S1. Frequency distribution of grain length and width in rice public dataset at NIAS Genebank. Frequency distribution of grain length (A) and grain width (B). Arrowheads indicate the trait data of BG23 and LG10 with measured place and year. (JPEG 612 kb) Additional file 2: Figure S2. Logarithm of odds (LOD) curves of five quantitative trait locus (QTL) analyses. LOD curves of QTL analyses for grain length are shown in A, C, E, G, and I. LOD curves of QTL analyses for grain length are shown in B, D, F, H, and J. F_2_ populations derived from Kasalath × BG23 (A and B), Kasalath × LG10 (C and D), Nipponbare × BG23 (E and F), Nipponbare × LG10 (G and H), and BG23 × LG10 (I and J) crosses. Dashed lines indicate LOD = 3.0 as threshold value. (JPG 541 kb) Additional file 3: Figure S3. Marker analysis of *GW2, GS3, qSW5/GW5,* and *GW8* alleles in Nipponbare, Kasalath, BG23, and LG10 lines. The results of marker analysis of *GW2* (A), *GS3* (B), *qSW5/GW5* (C), and *GW8* (D) alleles. Arrows indicate the larger grain (L) and smaller grain (S) alleles. (JPG 1337 kb) Additional file 4: Figure S4. Heat map for a two-dimensional genome scan with two-quantitative trait locus (QTL) models in F_2_ population derived from the BG23 × LG10 cross. The heat map of the maximum logarithm of odds (LOD) score. Upper left triangle: interaction (Full–Add) model. Lower right triangle: full model. Color-coded scales indicate the values on the left for the interaction model (LOD threshold = 5.35) and on the right for the full model (LOD threshold = 8.13). (JPG 879 kb) Additional file 5: Figure S5. Selection of segregation lines to evaluate the effect of quantitative trait loci (QTLs) detected on Chr3 and Chr6L. F_3_ lines with one segregating locus for Chr3 or Chr6L and three other fixed loci were selected. From these two lines, F_4_ plants homozygous for BG23 or LG10 were selected to evaluate the effect of QTLs on Chr3 and Chr6L. White and black boxes indicate BG23 and LG10 chromosomes, respectively. (JPG 287 kb) Additional file 6: Table S1. Primer sequences used for the quantitative trait locus (QTL) analysis of four populations. (XLSX 51 kb) Additional file 7: Table S2. Primer sequences employed in the quantitative trait locus (QTL) analysis and genetic mapping using the population from the BG23 × LG10 cross. (XLSX 43 kb) Additional file 8: Table S3. Primer sequences used in marker analysis. (XLSX 29 kb)

## Abbreviations

BS: Brassinosteroid
CAPS: Cleaved amplified polymorphic sequence
Chr6L: Long arm of Chr6
Chr6S: Short arm of Chr6
QTL: Quantitative trait locus
SEM: Scanning electron microscopy
SNP: Single nucleotide polymorphism
SSR: Simple sequence repeat
TRM: TONNEAU1 recruiting motif
