# Supplementary figures and images for: Detection of Novel QTLs Regulating Grain Size in Extra-Large Grain Rice (Oryza sativa L.) Lines
Source: Rice (N Y). 2016 Jul 25;9:34. doi: 10.1186/s12284-016-0109-2 (PMC4960101; doi:10.1186/s12284-016-0109-2)

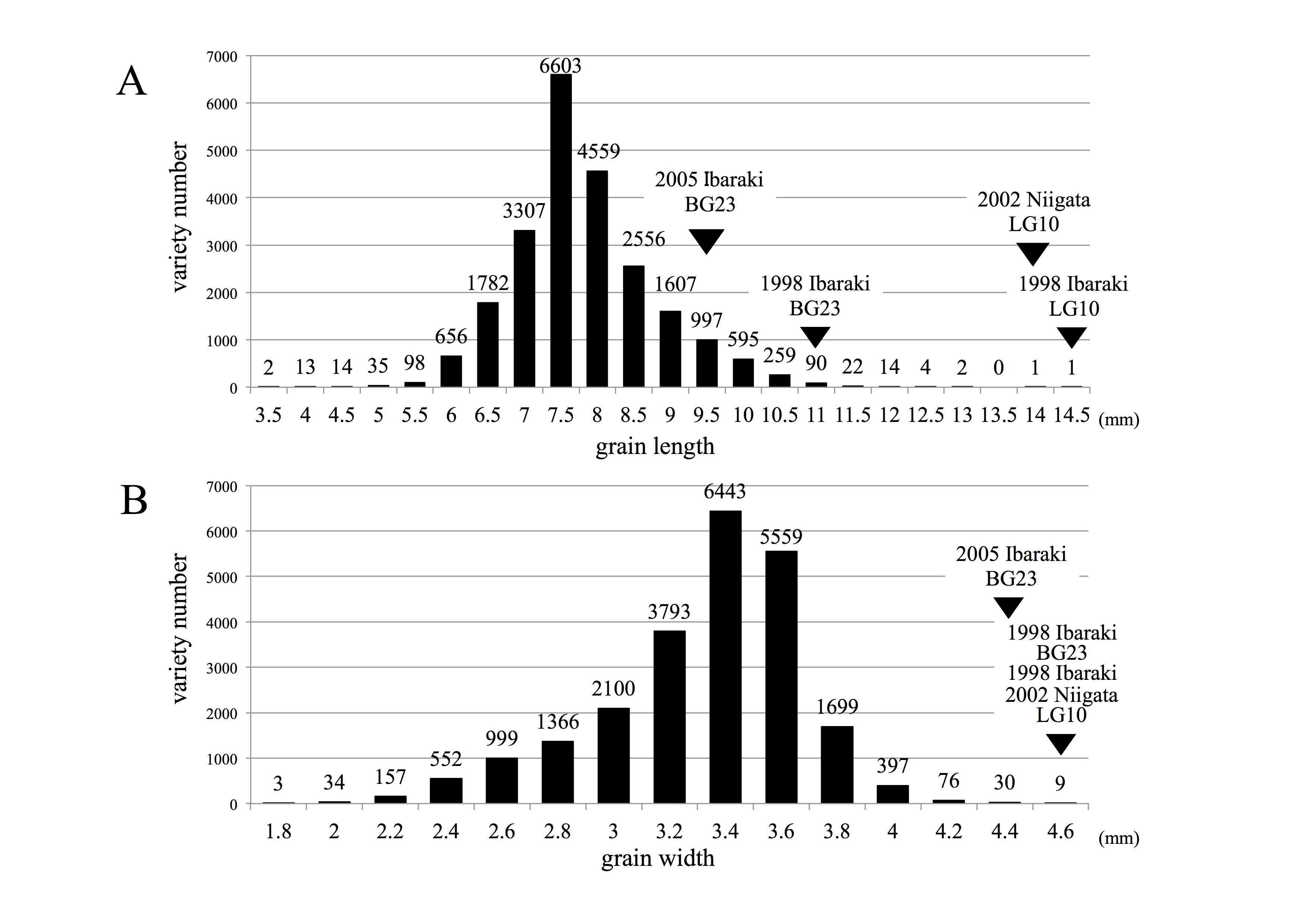

Supplement: Additional file 1: Figure S1. — Frequency distribution of grain length and width in rice public dataset at NIAS Genebank. Frequency distribution of grain length (A) and grain width (B). Arrowheads indicate the trait data of BG23 and LG10 with measured place and year. (JPEG 612 kb) [file 12284_2016_109_MOESM1_ESM.jpeg]

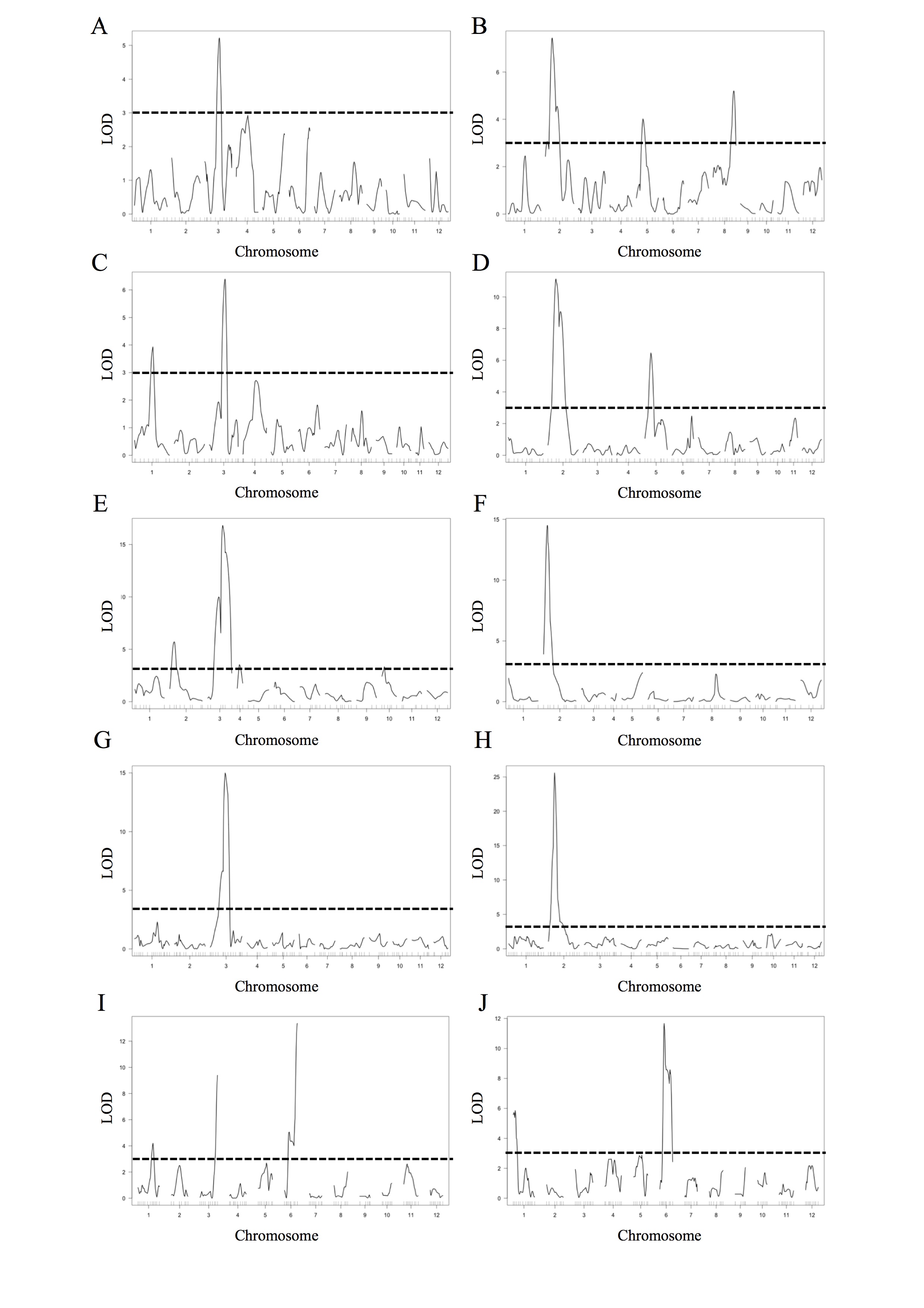

Supplement: Additional file 2: Figure S2. — Logarithm of odds (LOD) curves of five quantitative trait locus (QTL) analyses. LOD curves of QTL analyses for grain length are shown in A, C, E, G, and I. LOD curves of QTL analyses for grain length are shown in B, D, F, H, and J. F2 populations derived from Kasalath × BG23 (A and B), Kasalath × LG10 (C and D), Nipponbare × BG23 (E and F), Nipponbare × LG10 (G and H), and BG23 × LG10 (I and J) crosses. Dashed lines indicate LOD = 3.0 as threshold value. (JPG 541 kb) [file 12284_2016_109_MOESM2_ESM.jpg]

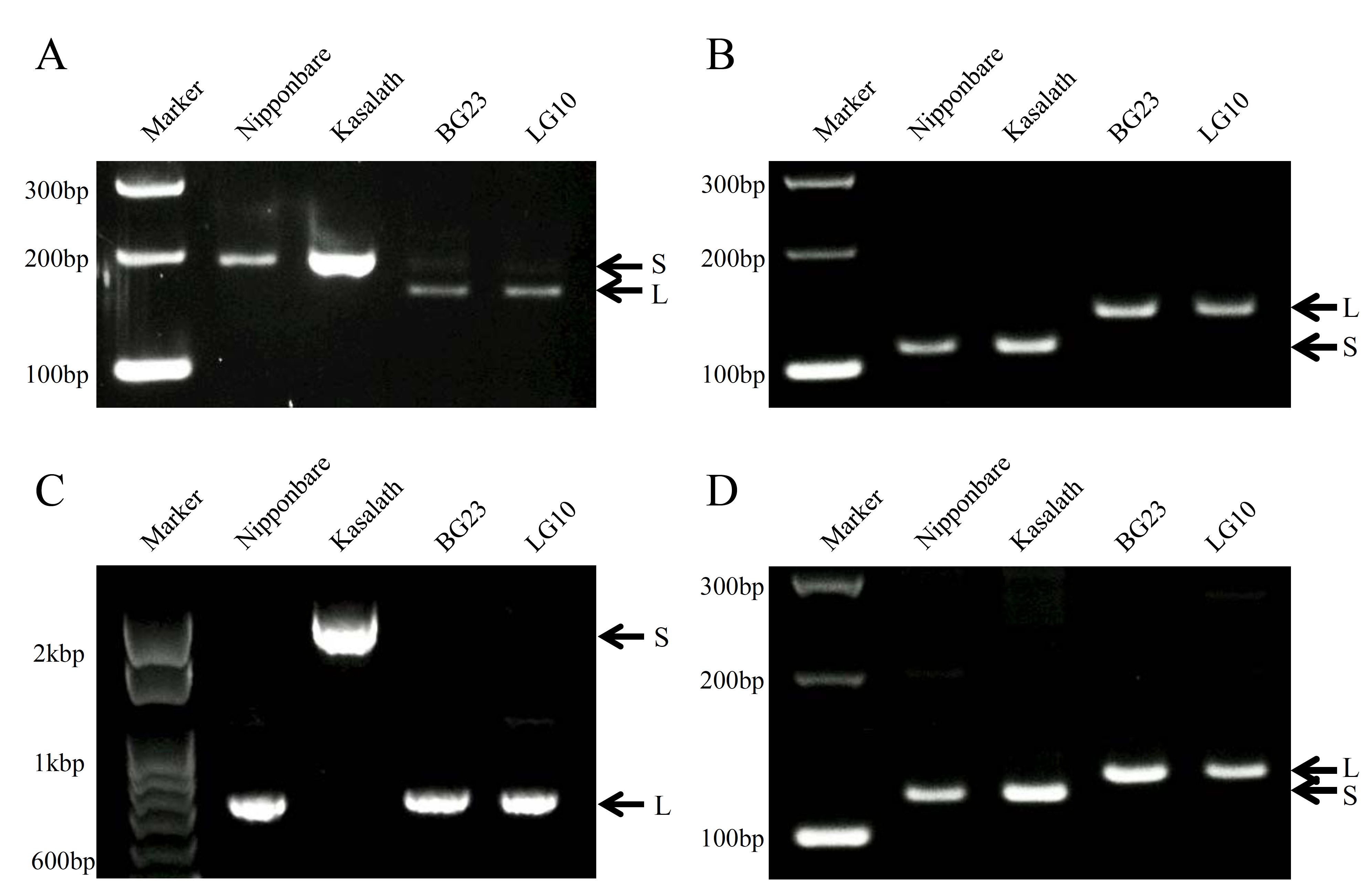

Supplement: Additional file 3: Figure S3. — Marker analysis of GW2, GS3, qSW5/GW5, and GW8 alleles in Nipponbare, Kasalath, BG23, and LG10 lines. The results of marker analysis of GW2 (A), GS3 (B), qSW5/GW5 (C), and GW8 (D) alleles. Arrows indicate the larger grain (L) and smaller grain (S) alleles. (JPG 1337 kb) [file 12284_2016_109_MOESM3_ESM.jpg]

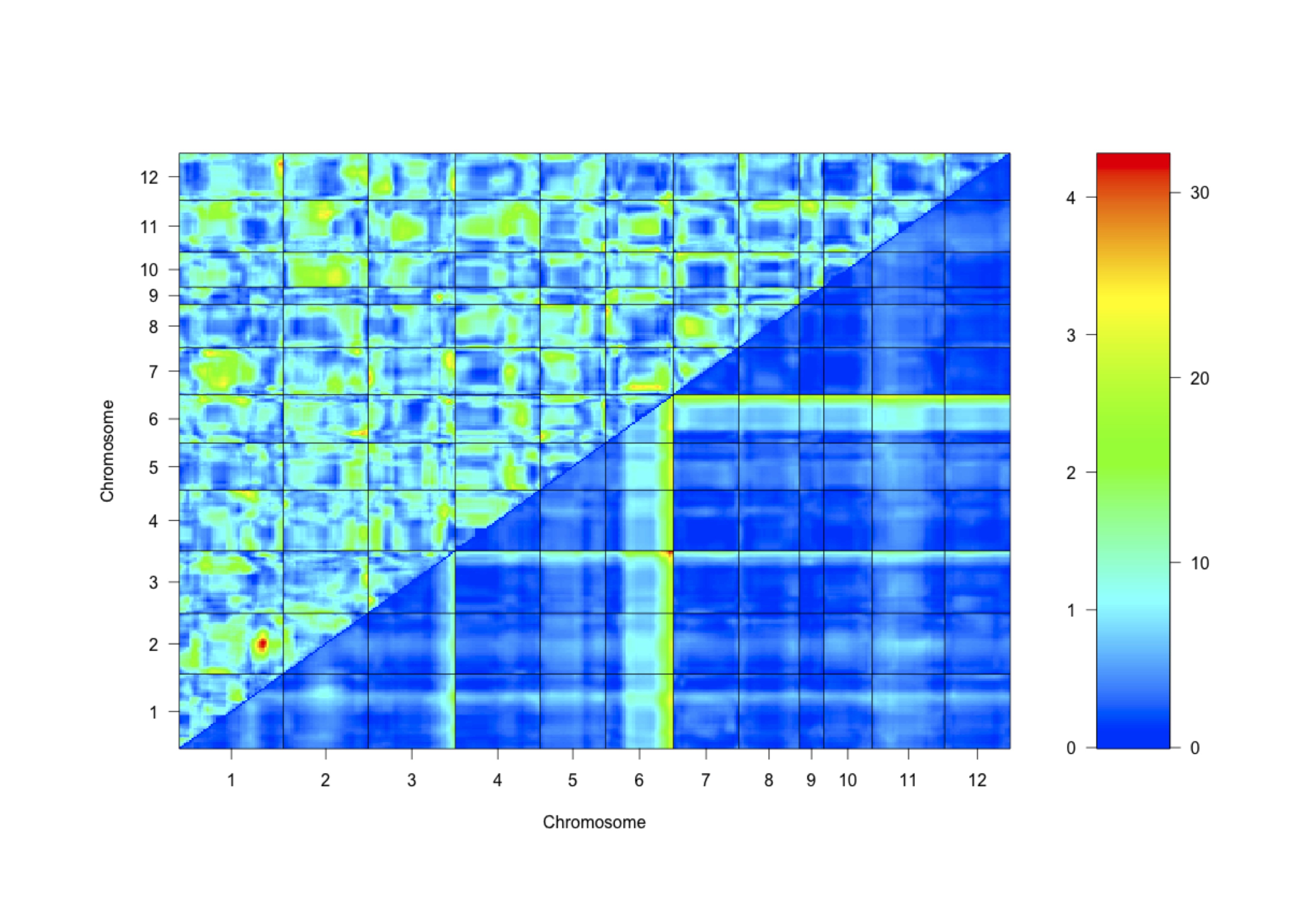

Supplement: Additional file 4: Figure S4. — Heat map for a two-dimensional genome scan with two-quantitative trait locus (QTL) models in F2 population derived from the BG23 × LG10 cross. The heat map of the maximum logarithm of odds (LOD) score. Upper left triangle: interaction (Full–Add) model. Lower right triangle: full model. Color-coded scales indicate the values on the left for the interaction model (LOD threshold = 5.35) and on the right for the full model (LOD threshold = 8.13). (JPG 879 kb) [file 12284_2016_109_MOESM4_ESM.jpg]

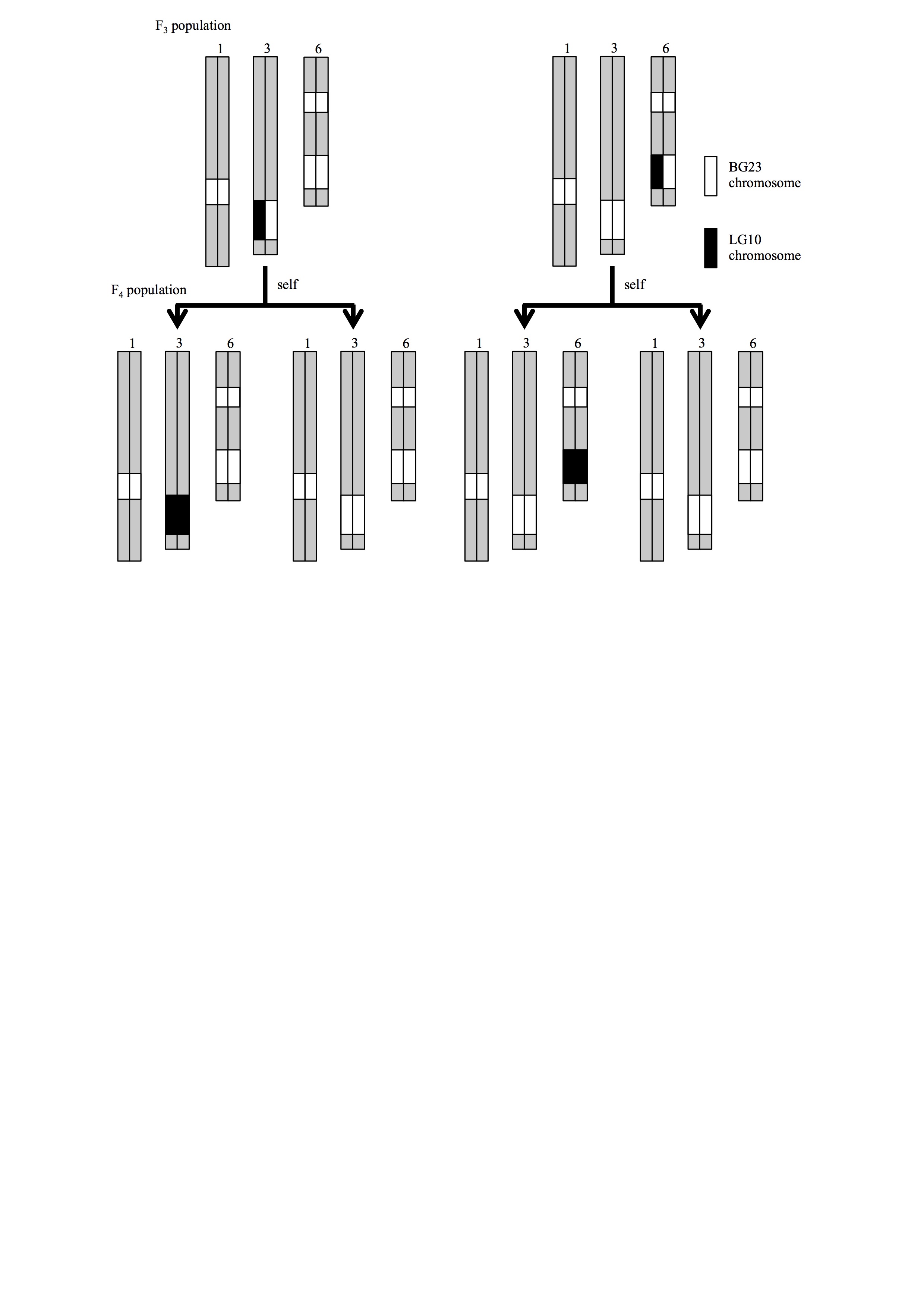

Supplement: Additional file 5: Figure S5. — Selection of segregation lines to evaluate the effect of quantitative trait loci (QTLs) detected on Chr3 and Chr6L. F3 lines with one segregating locus for Chr3 or Chr6L and three other fixed loci were selected. From these two lines, F4 plants homozygous for BG23 or LG10 were selected to evaluate the effect of QTLs on Chr3 and Chr6L. White and black boxes indicate BG23 and LG10 chromosomes, respectively. (JPG 287 kb) [file 12284_2016_109_MOESM5_ESM.jpg]
